# Succinylation-dependent mitochondrial translocation of PKM2 promotes cell survival in response to nutritional stress

**DOI:** 10.1038/s41419-018-1271-9

**Published:** 2019-02-20

**Authors:** Hailong Qi, Xianling Ning, Chang Yu, Xin Ji, Yan Jin, Michael A. McNutt, Yuxin Yin

**Affiliations:** 1 Institute of Systems Biomedicine, Peking University Health Science Center, Beijing, 100191 China; 2 Beijing Key Laboratory of Tumor Systems Biology, Peking University Health Science Center, Beijing, 100191 China; 3 Peking-Tsinghua Center for Life Sciences, Peking University Health Science Center, Beijing, China; 4 Department of Pathology, School of Basic Medical Sciences, Peking University Health Science Center, Beijing, 100191 China

## Abstract

Tumor growth and progression is characteristically associated with the synergistic effects of uncontrolled cellular proliferation and cell survival under stress. Pyruvate kinase M2 (PKM2) contributes to both of these effects. However, the specific mechanism by which PKM2 promotes uncontrolled proliferation or cell survival under stress in different nutritional environments is unclear. We show that succinylation mediated mitochondrial translocation of PKM2 under glucose starvation plays a role in switching the cellular machinery from proliferation to cell survival mode and vice versa. Mitochondrial PKM2 inhibits ubiquitination-mediated degradation of voltage-dependent anion channel 3 (VDAC3) and increases mitochondrial permeability to generate more ATP for cell survival under nutritional depletion. We found there is a positive correlation of upregulation of mitochondrial PKM2 and upregulation of VDAC3 in human colon cancer. This shows the mechanisms identified in this study in fact play a role in neoplastic biology. We therefore developed a small molecule designated compound 8 that blocks mitochondrial translocation of PKM2 and inhibits tumor development. Our data suggest that blocking PKM2 mitochondrial function with a small molecule inhibitor has potential for cancer treatment.

## Introduction

In the course of tumorigenesis, alterations in metabolism are the earliest observed difference which distinguishes cancer and normal tissues. These metabolic changes include the Warburg effect, which enables cancer cells to balance limited nutrition and rapid proliferation^1,2^. Evidence shows pyruvate kinase M2 (PKM2) contributes significantly to cancer metabolism and is important for aerobic glycolysis^3–6^. Pyruvate kinase is expressed in four isoforms in various tissues, and converts phosphoenolpyruvate (PEP) to pyruvate^7^. The isoforms PKL, PKR, and PKM1 are expressed mainly in normal tissues. However, PKM2 is preferentially expressed in embryonic tissues and in most kinds of cancer cells^8,9^. PKM2 exists in equilibrium between low- and high-activity states dependent on metabolic substrate mediated conformational change^10^. The allosteric regulation of PKM2 provides cancer cells with the flexibility to adapt to different microenvironments^11–14^. Posttranslational modification regulated nonglycolytic functions of PKM2 also play a role in the coordination of different microenvironments with cellular functions related to proliferation and cell survival^13,15–17^.

PKM2 has also been identified as a potential succinylation substrate of SIRT5^18^. A recent study indicates PKM2 is succinylated at K498, which affects reactive oxygen species in tumor cells^19^. It is intriguing that mitochondrial PKM2 regulates oxidative stress-induced apoptosis by stabilizing Bcl2^20^. However, whether SIRT5-mediated lysine de-succinylation regulates PKM2 function and thus plays a role in the regulation of mitochondrial function is unclear.

The voltage-dependent anion channel proteins (VDAC) are a small family of proteins that form an aqueous pore through the outer mitochondrial membrane, which allows exchange of metabolites. Three distinct VDAC isoforms are coded in humans^21^. In proliferating cells, VDACs which allow mediated fluxes of ATP/ADP and other respiratory substrates across the outer mitochondrial membrane balance oxidative phosphorylation and aerobic glycolysis to support energy requirements and biomass formation^22^.

In this study, we found PKM2 translocated into mitochondria and stabilizes VDAC3 in a succinylation dependent manner, which increases mitochondrial permeability. We identified a small molecule designed compound 8, which markedly reduces PKM2 activity. Compound 8 blocks the interaction of PKM2 and VDAC3, and blockage of PKM2 mitochondrial translocation by this molecule inhibits tumor growth in vivo.

## Results

### Glucose starvation promotes mitochondrial translocation of PKM2

**Fig. 1:**
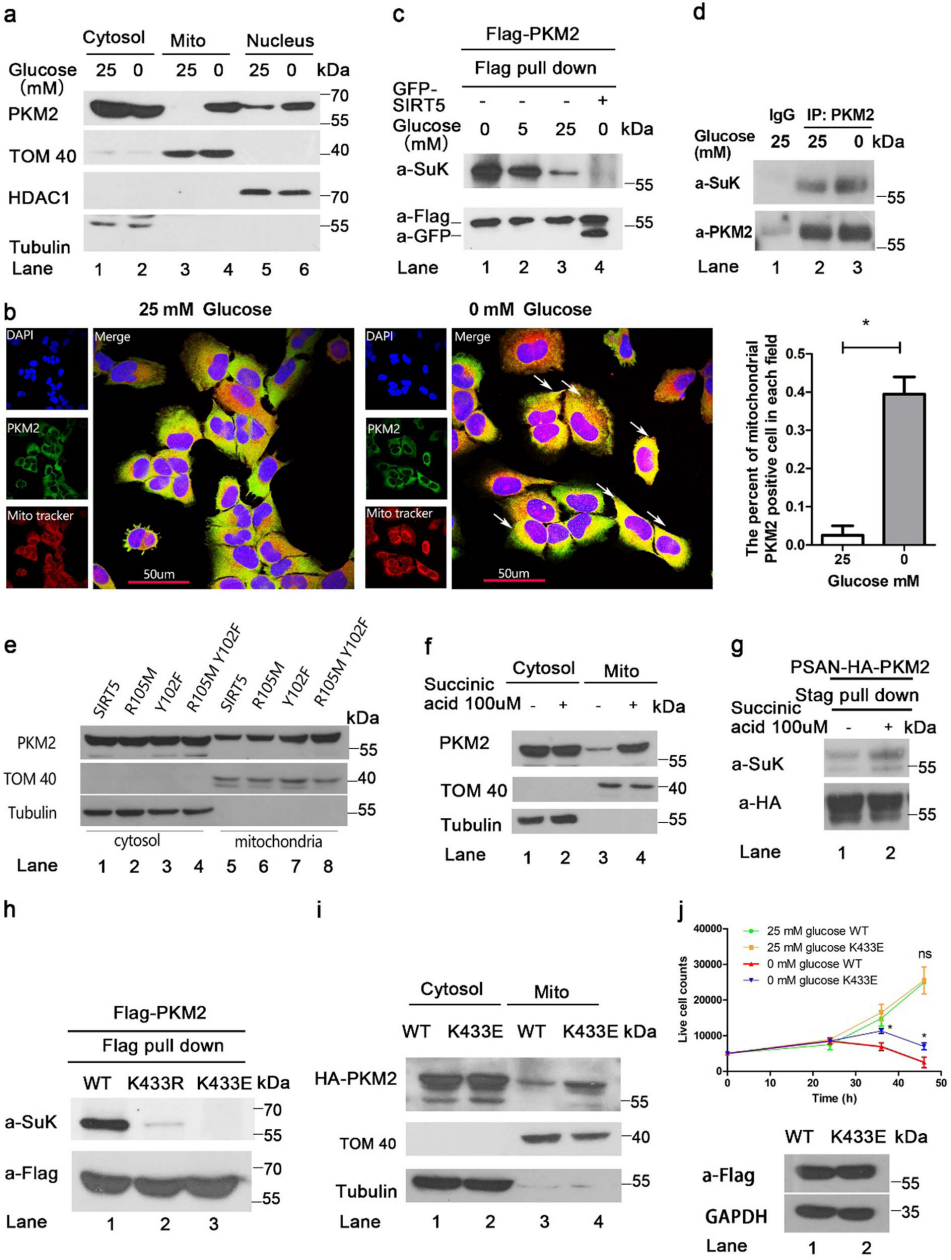
PKM2 localizes to mitochondria under glucose starvation. **a**, **b** PKM2 localizes to mitochondria under glucose starvation. Mitochondria and nuclear fractions were prepared from HCT116 cells under glucose starvation for 10 h. Immunofluorescence analysis was carried out after 10 h of glucose starvation. Mitochondria were identified with TOM40, nuclei were stained with DAPI, and an PKM2 monoclonal antibody was used to indicate endogenous PKM2. **c** Effect of glucose starvation on PKM2 succinylation in cells. Analysis of PKM2 succinylation in whole-cell extracts prepared from HCT116 cells treated with increasing concentrations of glucose (0, 5, and 25 mM) or SIRT5 overexpression. Lysates prepared from these cells were pulled down with Flag beads and analyzed with antibody to succinylated lysine. **d** Effect of glucose starvation on endogenous PKM2 succinylation in cells. Analysis of PKM2 succinylation in whole-cell extracts prepared from HCT116 cells treated with increasing concentrations of glucose (0 and 25 mM). Lysates prepared from these cells were enriched with PKM2 antibody and analyzed with antibody to succinylated lysine. **e** SIRT5 inhibits PKM2 mitochondrial translocation. HCT116 cells were transfected with expression constructs of GFP-tagged vector control GFP-SIRT5. After 10 h glucose starvation, western blot analysis of cytosolic and mitochondrial fractions was performed to evaluate translocation of PKM2 from the cytosolic compartment to the mitochondria. Tubulin and TOM40 were used as cytosolic and mitochondrial loading controls, respectively. **f** Succinic acids enhances PKM2 mitochondrial translocation. Mitochondrial and cytoplasmic fractions were prepared from HCT116 cells treated with 100 μM Succinic acid for 10 h. Western blot analysis of cytosolic and mitochondrial fractions was performed to evaluate translocation of PKM2 from the cytosolic compartment to the mitochondria. **g** Succinic acid enhances PKM2 succinylation. Analysis of PKM2 succinylation in whole-cell extracts prepared from HCT116 cells treated with or without 100 μM Succinic acid for 10 h. **h** Mutations K433R or K433E decreased PKM2 succinylation compared to WT PKM2. HCT116 cells were transfected with indicated plasmids, and PKM2 protein was immunoprecipitated followed by western analysis using either an anti-succinyl-lysine or anti-flag antibody to determine levels of succinylated and total PKM2 protein respectively. **i** Effect of mutation K433E on PKM2 subcellular localization. HCT116 cells transfected with HA-PKM2 (WT) or HA-PKM2 K433E were collected for cytosolic and mitochondrial fractionation followed by western analysis. The K433E mutation showed much more extensive localization to mitochondria as compared with WT. **j** K433E promotes cell survival under glucose starvation. 5000 HCT116 cells stably infected with Flag-PKM2 or Flag-PKM2 K433E virus were seeded into 96-well plates and live cell counts were measured in a blood cell counting chamber with trypan blue staining. The expression efficiency of K433E and WT PKM2 is equal

A previous study showed oxidative stress induces translocation of PKM2 to mitochondria and stabilizes Bcl2 to inhibit apoptosis^20^. Here, we sought to determine whether alterations in PKM2 mitochondrial translocation are coordinated with stimulation by environmental factors. Treatment of HCT116 cells with epidermal growth factor or insulin-induced or -inhibited nuclear translocation of PKM2 separately^13,15^, but did not increase PKM2 mitochondrial translocation (Supplementary Figure S1a, lanes 5, 6 vs. lane 4). On the other hand, glucose starvation resulted in mitochondrial accumulation of PKM2 as shown by western blot (Fig. 1a lane 4 vs. lane 3) and immunofluorescence analysis (Fig. 1b). Glucose starvation would be expected to cause an elevation of succinylaminoimidazolecarboxamideribose-5′-phosphate (SAICAR) with subsequent nuclear translocation of PKM2^4,23^. This raised the question as to whether SAICAR also mediates mitochondrial translocation of PKM2. Adenylosuccinate (ADSL) is an enzyme that converts SAICAR to AICAR to decrease cellular SAICAR, and knockdown of ADSL would therefore be expected to result in accumulation of SAICAR^4^. We observed that knockdown of ADSL (Supplementary Figure S1c) increased nuclear PKM2 without alteration in mitochondrial PKM2 (Supplementary Figure S1b, lane 4 vs. lane 3). These results demonstrate that the stimuli that drive translocation of PKM2 to the nucleus versus the mitochondria are distinctly different.

### Succinylation mediates mitochondrial translocation of PKM2

To gain insight into the mitochondrial translocation of PKM2 induced by glucose starvation, several posttranslational modifications, including acetylation, phosphorylation, and succinylation of PKM2 were evaluated under glucose starvation. Succinylation of PKM2 was significantly higher under glucose starvation (Supplementary Figure S1d, lane 2 vs. lane 1). Full-length human PKM2 was overexpressed in HCT116 cells, which were then treated with varying concentrations of glucose. Evaluation with a pan-succinyl lysine antibody showed the succinylated PKM2 band decreased as glucose concentration increased (Fig. 1c, lane 2, 3 vs. lane 1). As a positive control, co-overexpression of SIRT5 which is a PKM2 de-succinylase abolished succinylation of PKM2 (Fig. 1c, lane 4 vs. lane 1)^18,19^. Immunoprecipitation with PKM2 antibody showed that endogenous PKM2 of HCT116 cells was succinylated, and that this effect was enhanced by glucose starvation (Fig. 1d, lane 3 vs. lane 2). We also detected the succinylated PKM2 in other cancer cell lines, which indicated succinylation of PKM2 is extensive application for PKM2 regulation (Supplementary Figure S1e). We next investigated whether overexpression of SIRT5 decreased mitochondrial PKM2 under glucose starvation. Based on the study of Du et al.^24^, we constructed substrate interaction abolished mutants by changing Arg105 to Met or Tyr102 to Phe and dual mutation to Tyr102 and Arg105. These residues are important for binding succinyl groups and thus serve to confirm inhibition is SIRT5 de-succinylase dependent. We found that mutation of Tyr102 to Phe and dual mutation of SIRT5 failed to inhibit the mitochondrial translocation of PKM2 under glucose starvation compared to the WT SIRT5 (Fig. 1e, lanes 7, 8 vs. lane 5). Treatment of HCT116 cells with 100 μM succinic acid increased mitochondrial PKM2 (Fig. 1f, lane 4 vs. lane 3) consistent with increased PKM2 succinylation (Fig. 1g). These results argue that succinylation induces mitochondrial translocation of PKM2.

To specifically determine the primary site of PKM2 succinylation, several lysine residues which were previously identified by mass spectrometry (MS) and informatics prediction were replaced with arginine (R), which cannot be succinylated^19,25^. HCT116 cells were individually transfected, and succinylation of every mutant was tested. Substitution of the residue K433 significantly reduced the overall succinylation level of PKM2 compared to WT protein (Fig. 1h). The succinylation mimic mutation K433E enhanced mitochondrial translocation of PKM2, suggesting that K433 is the primary site of PKM2 succinylation responsible for mitochondrial translocation (Fig. 1i, lane 4 vs. lane 3). Conversely, although K498 succinylation decreased PKM2 activity^19^, the succinylation mimic mutation K498E had no effect on mitochondrial translocation (Supplementary Figure S1f, lane 4 vs. lane 3). PKM2 K433 is acetylated by P300 upon stimulation by oncogenic signaling, resulting in its translocation into the nucleus where it promotes cell proliferation, and K433 is also an important site for phosphotyrosine peptide binding. Moreover, K433E abolishes both nuclear translocation and phosphotyrosine peptide binding^26–28^. In view of the fact that under glucose starvation tumor cells would be expected to arrest proliferation to improve survival^29^. We, therefore, evaluated the effect of K433E on cell proliferation and survival in stable expression cell lines. The growth curves for cell line under glucose starvation in which K433E was stably overexpressed were significantly lower than the curves for cells with stable overexpression of WT PKM2, but cells with K433E overexpression showed longer survival (Fig. 1j). These data indicate succinylation mediated mitochondrial translocation of PKM2 is associated with cell proliferation and cell survival.

### PKM2 directly binds to and stabilizes VDAC3

**Fig. 2:**
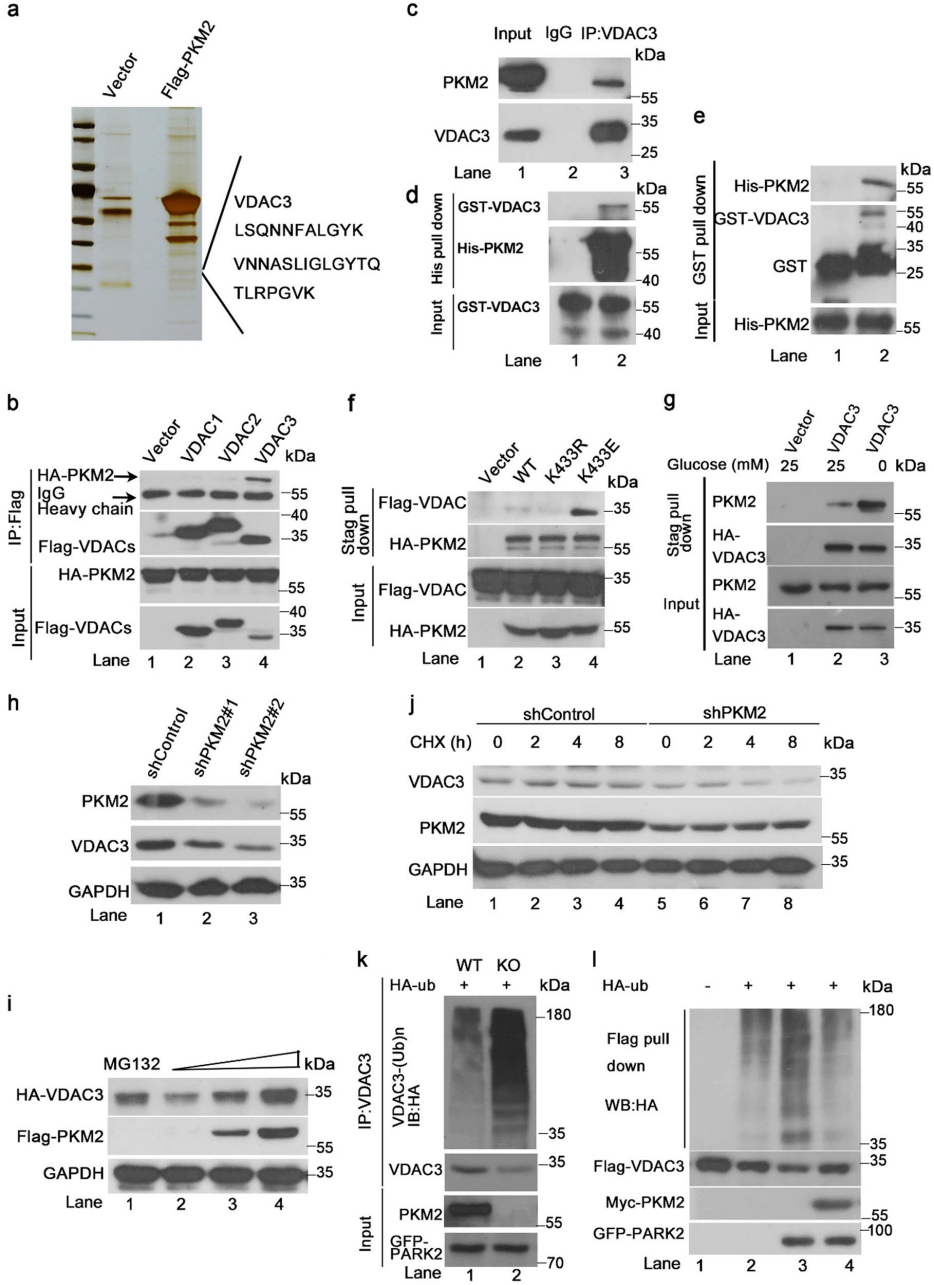
PKM2 directly binds to and stabilizes VDAC3. **a** VDAC3 peptide sequences were observed in the 31kDa band. Flag pull down and MS analysis of PKM2 associated proteins purified from transfected HCT116 cells. **b** Co-immunoprecipitation analysis showed that only VDAC3 binds directly to PKM2. **c** Western blot analysis of the endogenous interaction of PKM2 and VDAC3. HCT116 cell lysates were subject to immunoprecipitation with control IgG and anti-VDAC3 antibodies. **d**, **e** In vitro binding assay of purified His-tagged-PKM2 and GST-tagged-VDAC3. **f** K433E enhanced the interaction of PKM2 and VDAC3. Stagged-WT PKM2, a succinylation mimic K433E and a succinylation null mutant K433R and flag-VDAC3 were co-transfected into HCT116 cells for Stag bead pull down that specially pulls down Stagged proteins. **g** Glucose starvation enhanced the interaction of PKM2 and VDAC3. HCT116 cells were transfected with HA-tagged VDAC3, cells were harvested and subjected to Stag pull down assay after 8 h glucose starvation. **h** Western blot analysis of VDAC3 and PKM2 in HCT116 cell lysates after RNA-interference-mediated knockdown of PKM2. **i** Ectopic expression of Flag-PKM2 in HCT116 cells upregulates VDAC3 in a dose-dependent manner. Treatment with the proteasome inhibitor MG132 at 10 μM for 8 h also caused VDAC3 upregulation. **j** Expression levels of VDAC3 and PKM2 protein analyzed with immunoblotting in *PKM2* control (shControl) and *PKM2* knockdown (shPKM2). Cells were treated with cycloheximide (CHX, 150 µg/ml) at indicated times. **k** In vivo VDAC3 ubiquitination assay. Cellular level VDAC3 ubiquitination assays were carried out in PKM2 WT or knockout MEFs. Cells were co-transfected with His-HA-ubiquitin (Ub) and GFP-Parkin, immunoprecipitated with VDAC3 antibody and immunoblotted with antibodies to HA, VDAC3, PKM2, or GFP. Cells were treated with MG132 (10 μM) for 8 h before harvest. **l** In vivo VDAC ubiquitination assay. Cellular level VDAC ubiquitination assays were carried out in HCT116 cells. Cells were co-transfected with His-HA-ubiquitin (Ub), Flag-VDAC3, GFP-Parkin, and cMyc-PKM2 plasmids, immunoprecipitated with Flag beads and immunoblotted with antibodies to HA and Flag. Cells were treated with MG132 (10 μM) for 8 h before harvest

To determine how mitochondrial PKM2 promotes cell survival, Flag pull down assays coupled with MS was used to identify PKM2-interacting proteins (Fig. 2a). Outer mitochondrial membrane voltage-dependent anion channel family proteins (VDAC 1–3) were in the pull-down list. As VDACs affect outer mitochondrial membrane permeability, which regulates coupled respiration and cell survival^30^, we evaluated interaction of PKM2 and VDACs. Co-immunoprecipitation analysis of the three members of the VDAC family showed VDAC3 binds to PKM2 (Fig. 2b, lane 4 vs. lanes 2, 3). This interaction was further confirmed in vivo in two cancer cell lines (Fig. 2c, Supplementary Figure S2a) and in vitro (Figs 2d, e). Moreover, PKM1 showed almost no interaction with VDAC3 (Supplementary Figure S2b lane 2 vs. lane 3). However, the PKM2 succinylation mimic mutation K433E bound to VDAC3 more strongly than WT PKM2 (Fig. 2f, lane 4 vs. lane 2). Glucose starvation also elevated the interaction of VDAC3 and PKM2, which is in accords with increased succinylation level of PKM2 upon gluocose deprivation (Fig. 2g, lane 3 vs. lane 2). These results indicate binding of PKM2 to VDAC3 is enhanced by succinylation and glucose deprivation. We also mapped the sequences of VDAC3 for PKM2 interaction. We analyzed the structure of VDAC3 on the uniprot website and found that the amino acid sequences of VDAC3 are mainly trans-membrane. Only the N-terminus which may be exposed to the cytosol under certain circumstances as described in a previous study^31^, and the cytosolic sequences between two transmembrane sequences might serve as the PKM2 binding domain. Therefore, we constructed a series of VDAC3 deletions and found that deletions 49–54, 78–80, or 132–137 completely abolished interaction with PKM2 (Supplementary Figure S2c, lanes 4, 5, 7 vs. lane 2). As PKM2 has protein kinase function, we analyzed phosphorylation levels of VDAC3. There was no evidence of VDAC3 phosphorylation by PKM2 (data not shown). However, we found VDAC3 was downregulated when PKM2 was knocked down, and upregulated when PKM2 was overexpressed with no variation in its transcription level (Fig. 2h, lanes 2, 3 vs. lane 1, 2i, lanes 3, 4 vs. lane 2, Supplementary Figure S2d). Treatment with MG132 which is a proteasome inhibitor also upregulated VDAC3 (Fig. 2i, lane 1 vs. lane 2) suggesting PKM2 may interfere with proteasome degradation of VDAC3. A previous study demonstrated VDAC3 is ubiquitinated and degraded by Parkin, which is an E3 ligase^32^. To demonstrate PKM2 positively affects VDAC3 stability, cells were treated with the protein synthesis inhibitor cycloheximide. The half-life of VDAC3 was shortened in PKM2 knockdown cells (Fig. 2j). We, therefore, evaluated the ubiquitination level of VDAC3. Knockout of PKM2 enhanced ubiquitination of VDAC3 (Fig. 2k). Conversely, ectopic overexpression of PKM2 significantly inhibited ubiquitination of VDAC3 (Fig. 2l, lane 4 vs. lane 3). These results show PKM2 stabilizes VDAC3 through inhibiting ubiquitination dependent degradation.

### PKM2 and VDAC3 increase mitochondrial permeability under nutritional stress

**Fig. 3:**
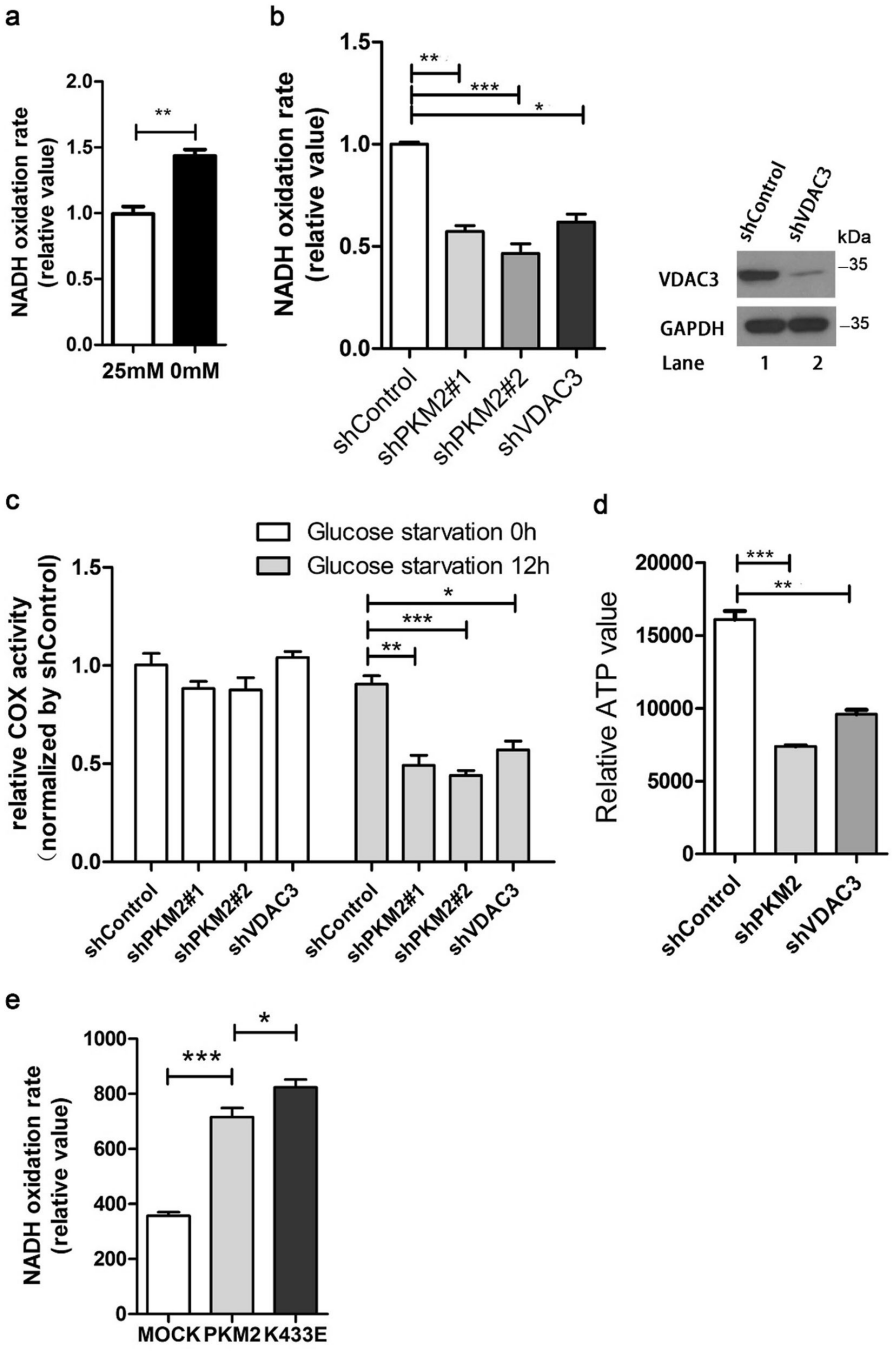
PKM2 increases outer mitochondrial membrane permeability and maintain mitochondrial activity upon nutrition stress. **a** Outer mitochondrial membrane permeability as determined by the NADH oxidation rate in isolated mitochondria from HCT116 cells after 0 or 25 mM glucose treatment for 6 h. **b** RNA-interference-mediated knockdown of PKM2 or VDAC3 caused a decrease in the rate of NADH oxidation. Mitochondria isolated from HCT116 cells were used for NADH oxidation assays. **c** Mitochondria from PKM2 and VDAC3 knockdown HCT116 cells were analyzed for COX activity after 12 h glucose starvation. Data are presented as mean ± SEM of three independent experiments. **d** PKM2 and VDAC3 maintain cellular ATP levels. ATP levels of indicated HCT116 cells were measured using the Cell Titer-Glo Luminescent Cell Viability Assay. **e** K433E enhances the NADH oxidation rate. Stagged-PKM2 and the succinylation mimic K433E were co-transfected into HCT116 cells for NADH oxidation rate assays

VDACs affect outer mitochondrial membrane permeability and cell survival. We found glucose starvation increased mitochondrial permeability^30^, which demonstrated mitochondrial membrane permeability correlates with cell survival under glucose starvation (Fig. 3a). We next evaluated mitochondrial permeability with or without PKM2 knockdown. The VDAC conductance state limits the passage of anionic metabolites and affects ATP/ADP exchange with the cytosol which will influence mitochondrial uptake of NADH^30^. Therefore, we determined the rate of NADH oxidation to evaluate purified mitochondrial permeability^30,33^. RNA-interference-mediated knockdown of PKM2 brought about a decrease in the rate of NADH oxidation in purified mitochondria (Fig. 3b). This suggested that the uptake of NADH for these mitochondria was reduced, which is consistent with the result of VDAC3 knockdown (Fig. 3b). Previous study shown that VDAC mediate rapid voltage-gated changes in membrane permeability, which could elevate or decrease overall mitochondrial activity^34^. Cytochrome c oxidation represents a critical feature of mitochondrial activity in coupling electron transport and oxidative phosphorylation. To demonstrate mitochondrial PKM2 and VDAC3 affect mitochondrial activity directly, we sought to determine whether knockdown of PKM2 and VDAC3 impaired mitochondrial respirasome by measuring the complex IV (cytochrome c oxidase, COX) activity. After 12 h glucose starvation, knockdown of PKM2 and VDAC3 decreased about 40% COX activity, which showed none significant differences in normal glucose medium (Fig. 3c). This result indicated that deficiency of PKM2 and VDAC3 impaired mitochondrial permeability and respiration. We thus investigated the ATP levels of PKM2 and VDAC3 knockdown cells under glucose starvation. Cellular ATP levels decreased after knockdown of PKM2 or VDAC3 (Fig. 3d). The PKM2 K433E succinylation mimic mutant increased mitochondrial PKM2, and we therefore compared NADH oxidation in HCT116 cells with K433E or ectopic overexpression of WT PKM2. In cells with K433E, oxidation of NADH was significantly more rapid than in cells with WT PKM2 (Fig. 3e). These results indicate mitochondrial PKM2 may increase mitochondrial permeability and ATP generation to support cell survival upon glucose starvation.

### PKM2 and VDAC3 promote cell survival under nutritional stress and tumor development

**Fig. 4:**
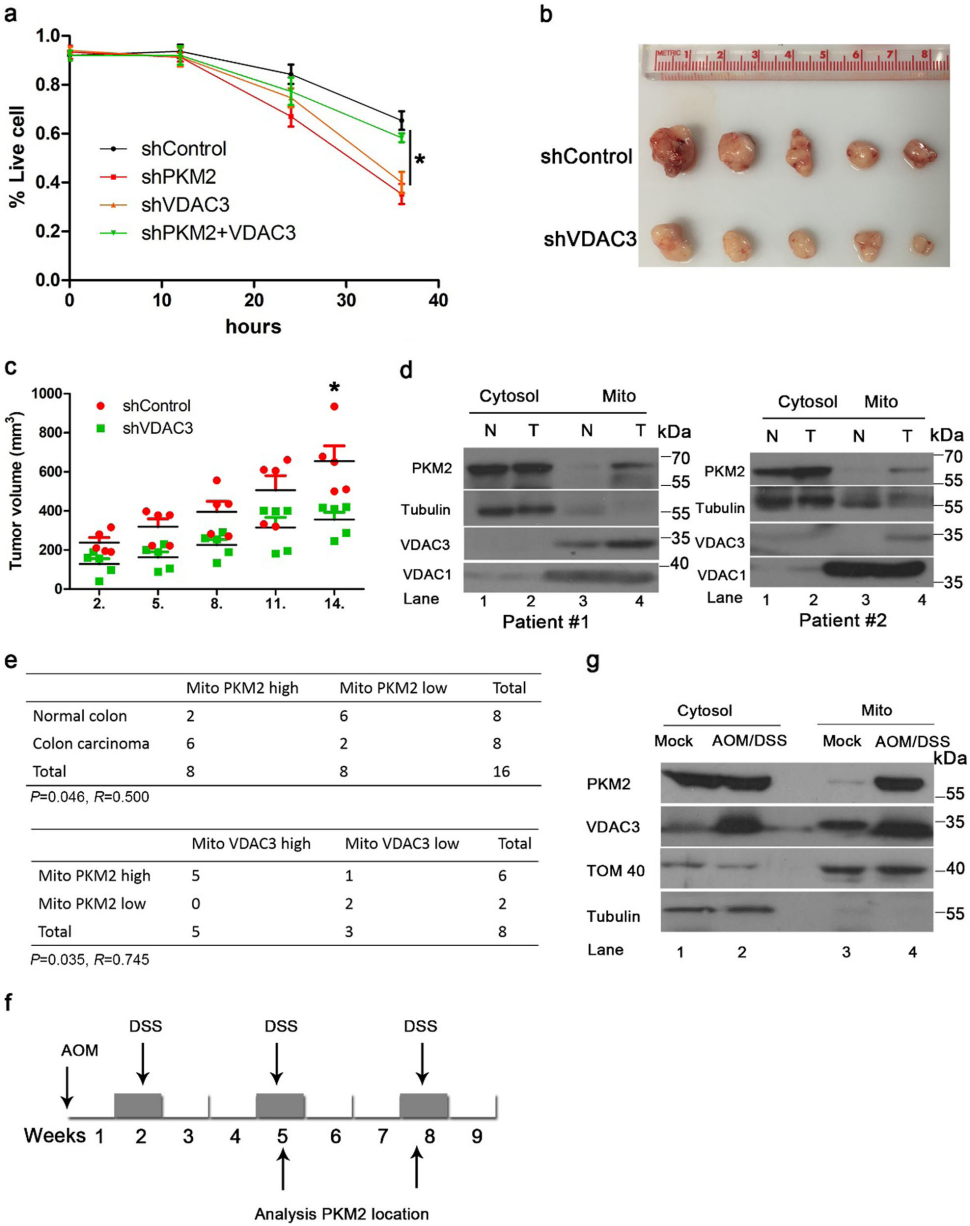
PKM2 and VDAC3 promote cell survival and tumor development under nutritional stress. **a** PKM2 and VDAC3 promote cell survival under nutritional stress. Survival of HCT116 cells in glucose-free media was measured with the Trypan blue exclusion method. **b**, **c** VDAC3 promotes tumor development. Tumor volume of VDAC3-kd HCT116 xenografts (*n* = 5). **d** PKM2 and VDAC3 are upregulated in human colon cancer. Fresh colon cancer and matched surrounding normal tissue from the same given patient were homogenized, and cytosolic and mitochondria fractions were separated. PKM2 was determined by western analysis. N (normal tissue), T (colon cancer). **e** Positive correlation of mitochondrial PKM2 and VDAC3 protein levels in human colon cancer. Statistical significance was determined with the *χ*^2^-test. *R* is the correlation coefficient. **f**, **g** Mitochondrial PKM2 and VDAC3 increased before solid tumor formation. **f** Sketch outlining the AOM/DSS-induced CRC mode. C57 mice were treated 1× with or without AOM, followed by periodic administration of 2% DSS in water. *n* = 12 per group. **g** Intestine issues were analyzed for mitochondrial PKM2 and VDAC3

We further investigated the effect of PKM2 and VDAC3 on cell survival and tumor development. VDAC3 knockdown sensitizes cells to glucose starvation induced death which is consistent with the result of PKM2 knockdown (Fig. 4a). Overexpression of VDAC3, but not overexpression of the interaction null mutants partially rescued the effect of PKM2 knockdown (Supplementary Figure S3a). We next investigated the effect of VDAC3 depletion on cell growth in xenograft tumor models in immunodeficient mice. VDAC3 knockdown suppressed growth of HCT116 tumor cells, which is in accord with the result of PKM2 knock down (Fig. 4b, c). To further determine whether the association of mitochondrial PKM2 with VDAC3 influences human cancer biology, mitochondria were isolated from human colon cancer and adjacent normal tissue. We observed upregulation of mitochondrial PKM2 in 6 of 8 tumor specimens and observed upregulation of VDAC3 in 5 of 8 cancer specimens as compared to adjacent nonneoplastic control tissues where neither of these molecules showed upregulation (Fig. 4d, Supplementary Figure S3b, c lane 4 vs. lane 3 for each patient, Supplementary Figure S3d). Increased levels of mitochondrial PKM2 and upregulated VDAC3 showed a significant positive correlation (*R* = 0.745, *P* = 0.035) in these colorectal carcinomas (Fig. 4e). As mitochondrial PKM2–VDAC3 contributes to tumor development, we investigated mitochondrial PKM2 and VDAC3 to determine whether they are upregulated in early stages of tumorigenesis in a mouse model. As the gastrointestinal tract has been reported to express PKM2 in adults^35^, an azoxymethane (AOM) and dextran sulfate sodium (DSS)-induced colorectal cancer mouse model was established to evaluate the translocation of PKM2 from the cytosol to the mitochondria. Mouse colon and rectum were evaluated at weeks 5 and 8 according to the AOM/DSS protocol (Fig. 4f). Mitochondrial PKM2 increased in the eighth week, and there was an associated increase in VDAC3 protein levels at that time (Fig. 4g, lane 4 vs. lane 3). No solid tumor was observed at these this time points. However, we observed solid tumor on the 80th day after induction, which is consistent with the typical course of AOM/DSS where tumor formation is generally observed after the 10th week^36^, which indicates that mitochondrial PKM2 and VDAC3 contribute to metabolic reprogramming in malignant transformation. These data suggest mitochondrial PKM2 and VDAC3 support metabolic reprogramming of tumor cells, and thereby defend against environmental stress during tumorigenesis. This raises the possibility colon cancer may be treated by specifically interfering with the PKM2–VDAC3 axis, and we next set out to screen small molecules for interference with the PKM2–VDAC3 axis.

### Identification and characterization of PKM2 inhibitors

A random screening of an in-house small molecule library (~1000 total molecules) was undertaken to identify new PKM2 modulators, which can block the interaction of PKM2 and VDAC3. The activity of these test compounds was assessed with a fluorescent PK-LDH coupled assay by a previously reported method (Supplementary Figure S4a)^37^. This resulted in the discovery of NP-1, which is a potent small molecule inhibitor of PKM2. We found NP-1 inhibitory activity was more powerful than shikonin, which is a reported PKM2 inhibitor (Supplementary Figure S4b)^38^. NP-1 displayed highly selective, dose-dependent inhibition of PKM2 with less inhibition of PKM1 and PKL (Supplementary Figure S4c). We undertook structural optimization by chemical modification. We designed new compounds utilizing a methyl group instead of the NP-1 chlorine moiety. Synthesis of target compounds was carried out according to the six-step protocol outlined in Supplementary Figure S4d, which was based on a previously described procedure^39,40^.

**Fig. 5:**
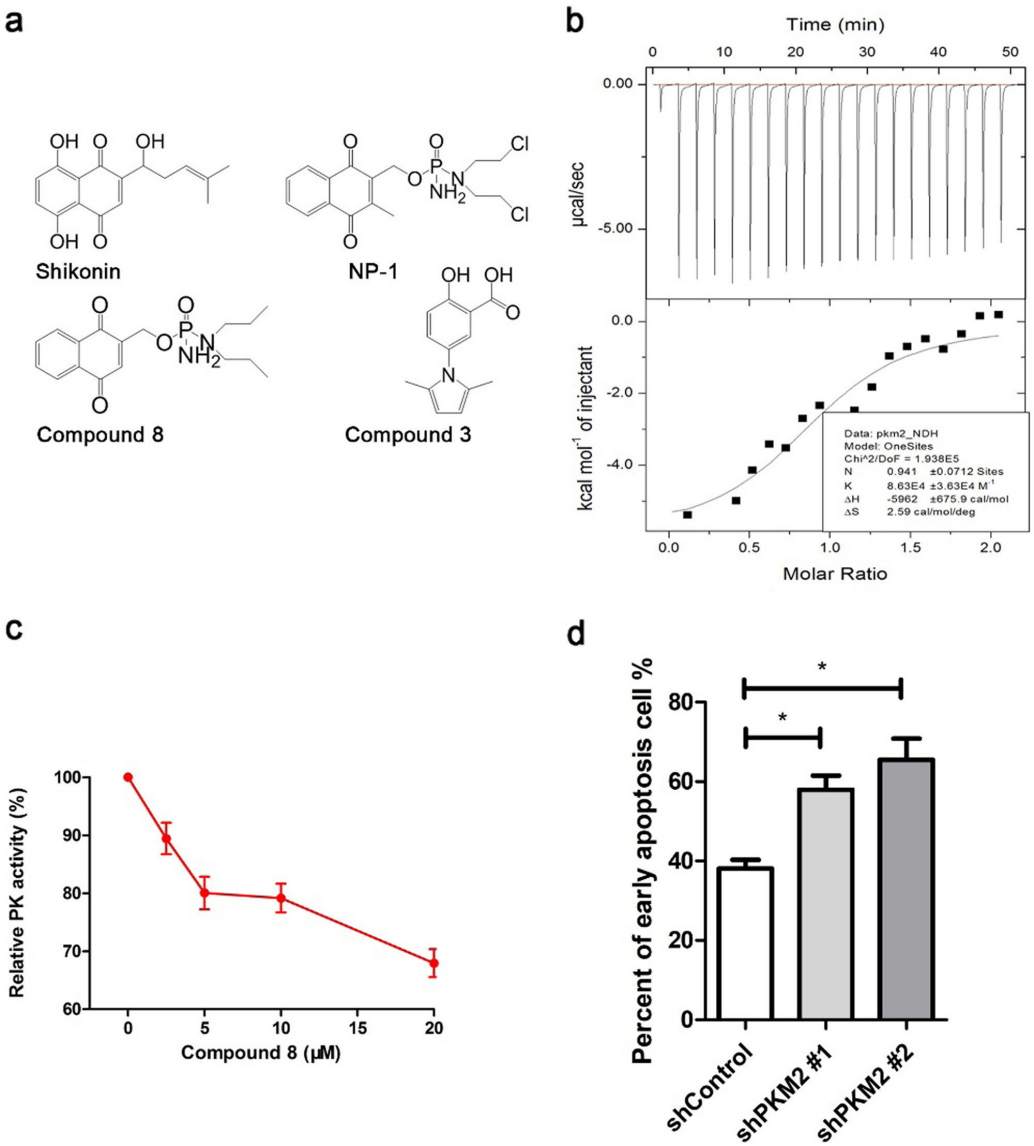
Identification and characterization of PKM2 inhibitors. **a** PKM2 inhibitors. Shikonin and compound 3 are known PKM2 inhibitors; NP-1 was the initial hit in the screen; Compound 8 is an NP-1 derivative with a higher level of PKM2 inhibitory activity. **b** Compound 8 binds to PKM2 with a KD value of about 11 µM measured by MicroCal iTC200. **c** Compound 8 inhibits PKM2 activity in HCT116 cells in a dose dependent manner. **d** Compound 8 increased the apoptosis of PKM2 knockdown cells. cells were harvested and subjected to annexin v stain assay after 12 h 20 µM compound 8 treatment

We tested modifications of target compounds for PKM2 inhibitory activity. Compound 8 (Fig. 5a) demonstrated the greatest PKM2 inhibitory activity (Table 1), with an IC50 of 0.77  μm.Evaluation of the structure–activity relationship (SAR) of compound 8 showed that the naphthoquinone unit is a pharmacophore which is crucial for inhibition of PKM2 activity, and substitution of a 3-methyl group for this naphthoquinone resulted in a 12-fold decrease in activity. Substitution of 3-H with naphthoquinone in compound 8 resulted in higher PKM2 inhibitory activity than that of the lead compound NP-1. The IC50 of compound 8 was about threefold lower than that of NP-1 (Table 1). We also evaluated the interaction of PKM2 and compound 8 with MicroCaliTC200, and determined that compound 8 binds PKM2 with a KD value of about 11 µM (Fig. 5b). These findings confirmed compound 8 is a PKM2 inhibitor of high selectivity, which is more potent than its antecedent compound NP-1.

**Table 1:** Evaluation of PKM2 inhibitory activity and selectivity of shikonin and target compounds

Compounds	*R*	*R*’	IC_50_ (PKM2)	IC_50_ (PKM1)	IC_50_ (PKL)
**8**	H	CH_3_	0.77 ± 0.30	10.48 ± 1.52	20.38 ± 3.54
**10**	CH_3_	CH_3_	13.93 ± 3.17	n.d.	n.d.
**NP-1**	CH_3_	Cl	2.93 ± 0.77	13.73 ± 1.14	69.62 ± 6.26
**Shikonin**			8.82 ± 2.62	12.96 ± 3.37	39.25 ± 6.53

*n.d.* not determined

We assessed the anti-tumor effects of compound 8 by evaluation of cancer cell viability. Compound 8 reduced cell viability with an IC50 of about 5 µM in MTT reduction assays in HCT116, MCF7, and H1299 cells (Table 2). We also found compound 8 inhibition of PKM2 activity in cancer cells was dose dependent (Fig. 5c). To determine whether cancer cell death brought about by compound 8 is PKM2 dependent, PKM2 was knocked down in HCT116 cells using two different shRNA sequences that specifically targeted exon 10 without interfering with PKM1 expression. Compound 8 is an inhibitor which causes a loss of function of PKM2. If PKM2 is a specially targeted by compound 8, its knockdown should render cells more vulnerable to compound 8-mediated PKM2 inhibition since shRNA and compound 8 work synergistically by reducing the intracellular drug target concentration. Downregulation of PKM2 expression partially enhanced cell toxicity of compound 8 as shown increased apoptotic cells in PKM2 knockdown cells (Fig. 5d). These results demonstrated that the effects of compound 8 on cancer cell viability are PKM2 dependent.

**Table 2:** Evaluation of target compound effects on viability of cancer cells (μM)

Compounds	IC_50_ (HCT116)	IC_50_ (MCF7)	IC_50_ (H1299)
**8**	5.19 ± 1.74	5.40 ± 1.14	4.75 ± 1.15
**10**	12.20 ± 2.24	9.70 ± 2.10	9.46 ± 1.86
**NP-1**	14.26 ± 3.57	17.51 ± 6.34	8.17 ± 1.92

### Compound 8 blocks PKM2 mitochondrial translocation under nutritional stress and inhibits tumor growth in vivo

**Fig. 6:**
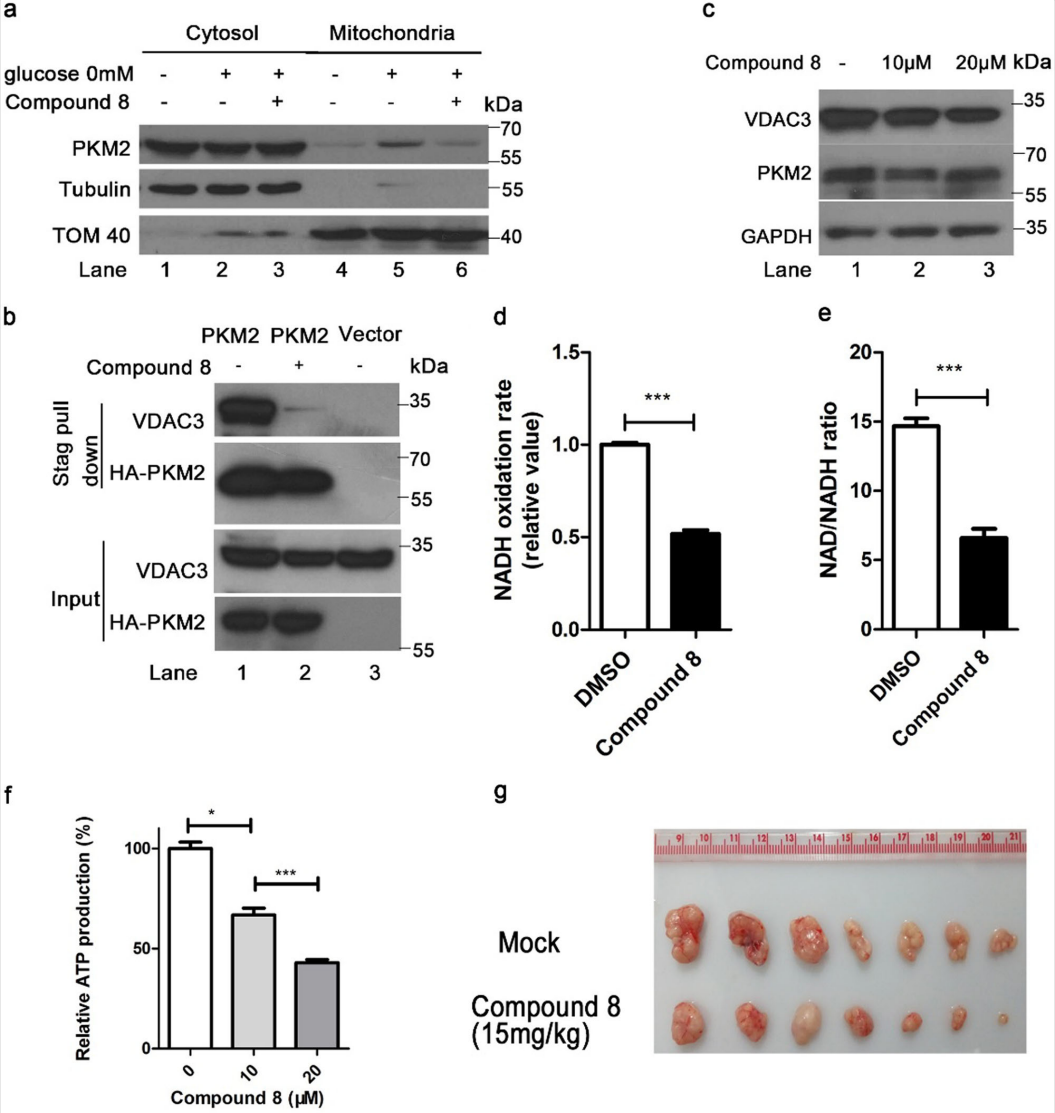
Compound 8 blocks PKM2 mitochondrial translocation under nutritional stress and inhibits tumor growth in vivo. **a** Compound 8 blocks glucose starvation induced PKM2 mitochondrial translocation. Isolation of mitochondria and immunoblot of PKM2, tubulin and Tom 40 were carried out with or without compound 8 treatment. **b** Compound 8 disrupts interaction of PKM2 and VDAC3. HCT116 cells were transfected with HA-tagged PKM2, cells were harvested and subjected to stag pull down after 8 h 20 µM compound 8 treatment. **c** Compound 8 induces VDAC3 for degradation. cells were harvested and evaluated VDAC3 protein levels after 8 h 10 or 20 µM compound 8 treatment. **d**, **e** Compound 8 caused a decrease in the rate of NADH oxidation. After 8 h of treatment with 20 µM compound 8, HCT116 cells mitochondria were isolated for NADH oxidation assays. NAD/NADH ratios in HCT116 cell lysates were evaluated by LC–MS. **f** Compound 8 inhibited ATP production in HCT116 cells. **g** The tumor volume (*n* = 7) of HCT116 xenografts from mice with intraperitoneal injection of compound 8 and vehicle control

We, therefore, sought to determine whether compound 8 interferes with the PKM2–VDAC3 axis. We demonstrated that 20 μM of compound 8 can restrain PKM2 mitochondrial translocation (Fig. 6a, lane 6 vs. lane 5). Compound 8 treatment abolished its interaction with VDAC3 (Fig. 6b, lane 2 vs. lane 1). As expected, compound 8 caused VDAC3 degradation (Fig. 6c). The rate of NADH oxidation in purified mitochondria from compound 8 treated cells was reduced (Fig. 6d, e). Compound 8 also inhibited cellular ATP production in a concentration-dependent manner (Fig. 6f). The anticancer properties of compound 8 were tested in xenograft cancer models to determine whether it inhibited tumor growth in vivo. Colon cancer xenograft growth was significantly reduced by treatment with 15mg/kg of compound 8 (Fig. 6g). These results suggest that compound 8 mediates a substantial decrease in mitochondrial PKM2 in cancer cells, which may offer a potential strategy for treatment of tumor. No similar findings have been reported with use of other PKM2 inhibitors.

## Discussion

PKM2 has been shown to play a central role in metabolic reprogramming during cancer progression. Tumor cells regulate PKM2 to support cell growth and survival by multiple pathways, which include PKM2 localization, expression, posttranslational modification, and allosteric regulation. In this study, we describe succinylation dependent mitochondrial translocation of PKM2 under glucose starvation. Mitochondrial PKM2 increases mitochondrial permeability by specially stabilizing VDAC3, which contributes to cell survival under glucose starvation. This serves to explain the upregulation of mitochondrial PKM2 and VDAC3 in human colon cancer and to show how these molecules promote tumor development in vivo. In mammals, three isoforms (VDAC1, VDAC2, and VDAC3) have been identified^41–44^. Compared to VDAC1 and VDAC2, cysteines of VDAC3 are more preferentially exposed to the mitochondrial intermembrane space and can be oxidized regulation which may distinguish VDAC3 function from other isoforms^45,46^. Under glucose starvation, enhanced mitochondrial respiration may alter the VDAC3 oxidation status and mediate the specific PKM2 interaction which need to be further investigated. Among the three PKM2 interacted cytosol-exposed loops of VDAC3, the amino acids 132–137 showed the least identity to VDAC1 and VDAC3, which may also responsible for the specific PKM2 interaction.

PKM2 regulation offers several potential strategies for targeting PKM2 to treat cancer, such as activating or inhibiting pyruvate kinase, and blocking PKM2 protein kinase activity. Many PKM2 activators have been identified in recent years. However, only a small number of PKM2 inhibitors have been reported, and their mechanisms of action are unclear. Compound 3, which inhibits PKM2 activity at micromolar concentrations was identified in a high-throughput screen, but this agent shows little suppression of tumor cell proliferation below 200 µM^37^. Shikonin inhibits PKM2 and induces cell apoptosis at low concentrations, but shikonon is a multitarget drug which hinders further study of its mechanism^38^. In this study, we identify a novel chemical class of PKM2 inhibitors. Compound 8 inhibits PKM2 response, and has a selectivity which is greater than compound 3 or shikonin which is regarded as the most potent PKM2 inhibitor reported to date. Moreover, compound 8 induces tumor cell death at a low concentration.

We performed binding pocket exploration and docking to predict the binding site of compound 8. Our findings raised the possibility compound 8 inhibits PKM2 by binding at the same site as phenylalanine^47^. However, mutation of amino acids around this binding site did not influence the inhibitory activity of compound 8, and it is therefore possible that there is an as yet unidentified site where inhibitory activity is regulated. Further investigation will focus on structural studies of the compound 8 complex, which may lead to identification of compounds with higher inhibitory activity and selectivity.

## Materials and methods

### Purification of recombinant pyruvate kinase isoforms and VDAC3

Human cDNA for PKM2, PKM1, and VDAC3 were separately cloned into a pET28a (+) and pGEX-4T-1 vector with an N-terminal His-tag and N-terminal GST-tag and purified from the *Escherichia coli* strain BL21 (Invitrogen) using Ni–agarose beads and GST agarose beads (Qiagen) as described previously^7^. PKL was purchased from Sino Biological Inc. in China.

### PKM2 activity assay

Pyruvate kinase activity was measured with a fluorescent pyruvate kinase–lactate dehydrogenase coupled assay as described previously. All compounds were tested in a kinetic mode by coupling the generation of pyruvate by pyruvate kinase to the depletion of NADH through lactate dehydrogenase. For PKM2, 40 μL of buffer (50 mM Tris–HCl, pH 7.5, 10 mM KCl, 5 mM MgCl_2_), 1 μL of compound and 5 μL of enzyme solution were dispensed into Corning black solid 96-well plates and incubated for 15 min. Totally, 55 μL of substrate mix (final concentration, 0.5 mM PEP, 4.0 mM ADP, 0.12 mM NADH, 0.25 mM FBP, and 1 unit LDH) were then added, and the plates were placed in a FlexStation3 (Molecular Devices), followed by determination of NADH fluorescence at 30 s exposure intervals for 3–6 min. Data were collected on a FlexStation3.

HCT116 cells were seeded overnight into 6-well plates at 1,000,000 cells/well in DMEM media. Test compounds were added for 6 h. Cells were washed two times with ice-cold phosphate-buffered saline (PBS) and lysed in lysis buffer. Lysate was analyzed for pyruvate kinase activity as described above.

### Cell culture

Cells were cultured in Dulbecco’s modified Eagle’s medium (DMEM) supplemented with 10% FBS at 37 °C in 5% CO_2_. The human cancer cell line HCT116 was obtained from the American Type Culture Collection (ATCC) in 2009 and maintained according to ATCC recommendations. This cell line was authenticated by Beijing Micro read Genetics in November 2016 using STR profiling. For low glucose stimulation, HCT116 cells were incubated in medium without glucose for 10 h.

### Cell viability test

Cells were seeded in 96-well plates at 5000 cells/well in DMEM media overnight. Cells were treated with different concentrations of test compound for 48 h at 37 °C in 5% CO_2_. Cell viability was assayed with the MTS assay (Promega) and trypan blue staining according to the manufacturer’s instructions.

### shRNA constructs and lentiviral production

Two shRNAs with PKM2 specific exon 10 sequence knockdown were used (5'-CCGGGGGTGAACTTTGCCATGAATGCTCGAGCATTCATGGCAAAGTTCACCCTTTTTG-3'; 5'-CCGGTCATTGCTGTGACCCGGAATCCTCGAGGATTCCGGGTCACAGCAATGATTTTTG-3'), and a shRNA with no effect on PKM2 levels was employed as a control (5'-CCGGGAGGCTTCTTATAAGTGTTTACTCGAGTAAACACTTATAAGAAGCCTCTTTTTG-3). Lentivirus was packaged using a three-plasmid system. ShRNAs were inserted into the pLKO.1-puro vector and co-transfected into 293T cells together with PAX2 and PMD2G. Lentivirus was harvested 36 h after transfection.

### Isolation of mitochondria

HCT116 cells were harvested and washed two times with cold PBS. Cells were then homogenized in a buffer (220 mM mannitol, 70 mM sucrose, 2 mM EGTA, 5 mM MOPS-Tris pH 7.4, 2 mM Taurine, 0.2% BSA). The homogenate was spun for 5 min at 800*g*.The supernatant containing the cytoplasmic and mitochondrial fractions were transferred to a fresh tube and spun for 5 min at 12,000*g*. The pellet contained the mitochondrial fraction and the supernatant contained the cytoplasmic fraction.

### NADH oxidation assay

NADH oxidation in the mitochondria was measured by resuspending mitochondria isolated from HCT116 cells after knockdown or overexpression of PKM2, K433E, K433R, PKM1, or treatment with 20 μM compound 8 for 8 h in 25 mM NADH. Absorbance was monitored at *λ* = 340 nm. Hypotonically shocked mitochondria were run as a control in parallel assays for mitochondrial intactness.

### Co-immunoprecipitation assay

About 2.5 mg whole cellular extracts were prepared in 700 μl RIPA buffer (50 mM Tris-HCl, pH 8.0, 150 mM NaCl, 1.5 mM MgCl_2_, 0.1% sodium dodecyl sulfate (SDS), 0.5% deoxycholate, 0.5% NP-40, 1 mM phenylmethylsulfonyl fluoride (PMSF), 1× protease inhibitor cocktail (Roche)). A 4 μl of the mouse VDAC3 antibody and preimmune serum (homemade) were added into whole cellular extracts and incubated 3 h at 4 °C, followed by addition of 20 μl protein A/G agarose (Santa Cruz, sc-2003; only for IP with unconjugated antibodies noted above) for 1 h. Then samples were spun for 1 min at 2200*g* and added RIPA buffer diluted with PBS (1:9) for washing three times. The binding components were eluted by boiling with 2× SDS-polyacrylamide gel electrophoresis (PAGE) loading buffer, and were analyzed with western blot in 10% SDS-PAGE gel. PKM2 (D78A4) XP^®^ Rabbit mAb (#4053, CST) and VDAC3 Rabbit pAb (bs-7647R, Bioss) were used to blot PKM2 and VDAC3 separately. For Flag or S-tag pull down, 20 μl Flag M2 Affinity Gel (Sigma, A2220), or S-protein agarose (EMD Millipore Corp., Billerica, MA USA, 69704–4) were added into whole cellular extracts and incubated 3 h at 4 °C. After washing with RIPA buffer diluted with PBS (1:9) three times, the binding components were eluted by boiling with 2× SDS-PAGE loading buffer, and were analyzed with western blot.

### GST-VDAC3, His-PKM2 protein, and in vitro pulldown assay

GST-VDAC3 and His-PKM2 were prepared by bacterial purification. For His-pulldown assays, 2.5 μg of His or 2.5 μg of His-PKM2 was incubated with in vitro translated GST-VDAC3,and the solution was incubated with Ni–NTA agarose. Proteins were eluted and resolved on an 8% SDS-PAGE gel for Western blot analysis using anti-GST or anti-His antibody.

### Measurement of ATP

HCT116 cells were cultured in DMEM containing 10% FBS and seeded into six-well plates. Twenty-four hour after seeding, the medium was changed to DMEM containing 10 or 20 μM compound 8 for 8 h. ATP levels were measured using the Cell Titer-Glo Luminescent Cell Viability Assay (Promega).

### Western blotting

Cells were lysed in buffer containing 50 mM Tris pH8.0, 2 mM EDTA, 150 mM NaCl, 0.5% Nonidet P-40, 10% glycerol and protease inhibitor PMSF. Western blot analysis was carried out based on standard methods. The following commercial antibodies were used as probes: anti-Flag antibody (Sigma, F3165), anti-HA antibody (Sigma, H3663), anti-α-Tubulin (EasyBio, BE0026–100), anti-PKM2 (RuiYingBio, RLT3777), anti-TOM 40 (Santa Cruz sc-365467), anti-succinyllysine antibody (PTM BIO PTM-901), anti-GAPDH (SanJian).

### RT-qPCR

Following treatment with compound 8 (20 μM) for 8 h or knockdown of ADSL, PKM2, or VDAC3 were screened with puromycin for 5 days, and total HCT116 cell RNA was extracted using TRIzol (Invitrogen). cDNA synthesis was carried out using a cDNA synthesis kit (Tansgene). Quantitative PCR (qPCR) then was carried out using SYBR Green Master Mix (Transgene) in a bio-red Real-time PCR machine.

### Xenograft experiments

All animal procedures were performed in accordance with guidelines approved by the Animal Experimentation Ethics Committee of Peking University. Female nude mice (BALB/C, 4–6 weeks old) were injected with 2.0 × 106 HCT116 cells subcutaneously in the armpit. Approximately, 10 days later, pairs of mice (*N* = 7) were injected with compound 8 or vehicle control. Intraperitoneal injection was performed in the mouse. The animals were injected once every two days. Tumor measurements were performed once every 2 days. Tumor volume was calculated using the following equation: *V* = L(S2)*π*/6, where L is the longer and S is the shorter of the two tumor dimensions.

### Chemistry

Chemical synthesis and characterization is provided in the Supplementary information.

### Statistical analysis

The statistical significance of differences between various groups was calculated with the two-tailed paired *t* test, and error bars represent standard error of the mean (SEM). Statistical analysis, unless otherwise indicated, was performed using GraphPad Prism 5. *P* values of less than 0.05 were considered statistically significant. ^∗^*P* < 0.05, ^∗∗^*P* < 0.005, and ^∗∗∗^*P* < 0.001. Data are shown as mean ± SEM.

## Supplementary information

Supplementary figures and methods
