# Supplementary material for: Succinylation-dependent mitochondrial translocation of PKM2 promotes cell survival in response to nutritional stress
Source: Cell Death Dis. 2019 Feb 20;10(3):170. doi: 10.1038/s41419-018-1271-9 (PMC6382874; doi:10.1038/s41419-018-1271-9)
Supplement: Supplementary file 1 — Supplementary figures and methods [file 41419_2018_1271_MOESM1_ESM.docx]

**Supplementary Information**


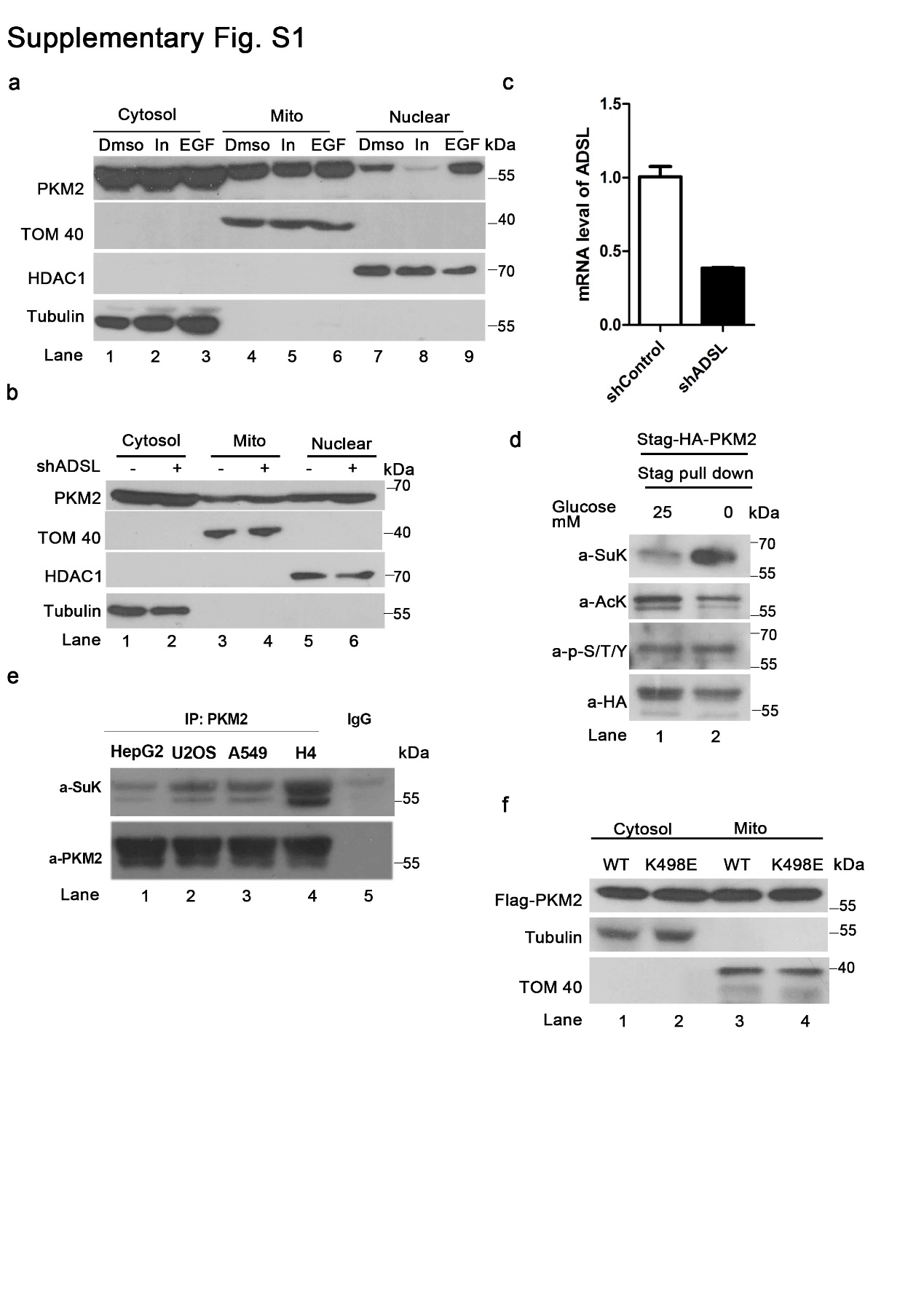


Supplementary Fig. S1. Succinylation mediates mitochondrial translocation of PKM2.

**a** EGF and insulin do not increase mitochondrial PKM2. Mitochondria and nuclei were isolated from HCT116 cells after indicated treatment and PKM2 protein was evaluated in cytosolic, mitochondrial and nuclear fractions. **b** and **c** Saicar does not induce mitochondrial PKM2 elavation. ADSL mRNA levels were evaluated in HCT116 cells after PLKO-mediated knock down (c) and cells were subjected to isolation of mitochondria and nuclei to detect PKM2 protein (b). **d** The phosphorylation, acetylation and succinylation modification of PKM2 after 10 hours of glucose starvation treatment. Isolated cell lysates were subjected to PKM2 immunoprecipitation and stained with indicated antibody. **e** Confirm the succinylation of PKM2 in different cancer cell lines. Succinylated PKM2 levels in A549 (lung cancer, lane 3), H4 (glioma, lane 4), HepG2 (hepatocellular carcinoma, lane 1) and U2OS (osteosarcoma, lane 2) were evaluated. **f** Effect of mutation K498E on PKM2 subcellular localization. HCT116 cells transfected with HA-PKM2 (WT) or HA-PKM2 K498E were collected for cytosolic and mitochondrial fractionation followed by western analysis. The K498E mutation showed localization to mitochondria which was not more extensive than that with WT PKM2.


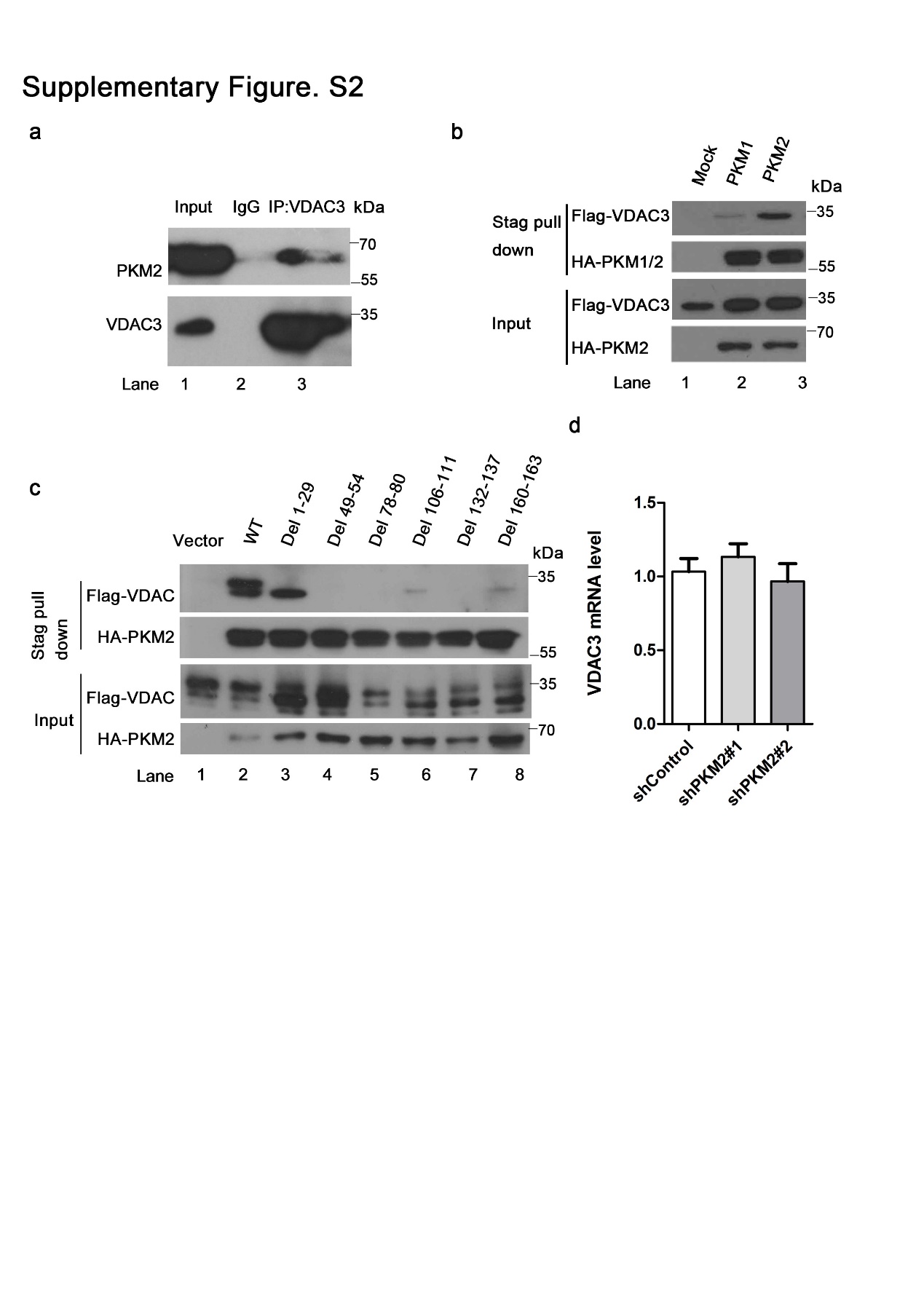


Supplementary Fig. S2. PKM2 directly binds to and stabilizes VDAC3.

**a** *In vivo* PKM2 and VDAC3 binding. Endogenous VDAC3 were immunoprecipitated from glioma H4 cell line and immunoblotted with antibodies against VDAC3 and PKM2. **b** Stag-HA-PKM1, PKM2 and Flag-VDAC3 were co-transfected into HEK293T for Stag pull down. PKM1 showed almost no evidence of interaction with VDAC3. **c** Identification of PKM2 and VDAC3 binding sites. HEK293T cells were co-transfected with Stag-HA-PKM2 and Flag-tagged full-length VDAC3 or its deletion mutants followed by immunoprecipitation with S-beads and immunoblotting with antibodies against HA or Flag. **d** RNA-interference-mediated knockdown of PKM2 had no effect on the VDAC3 mRNA level.


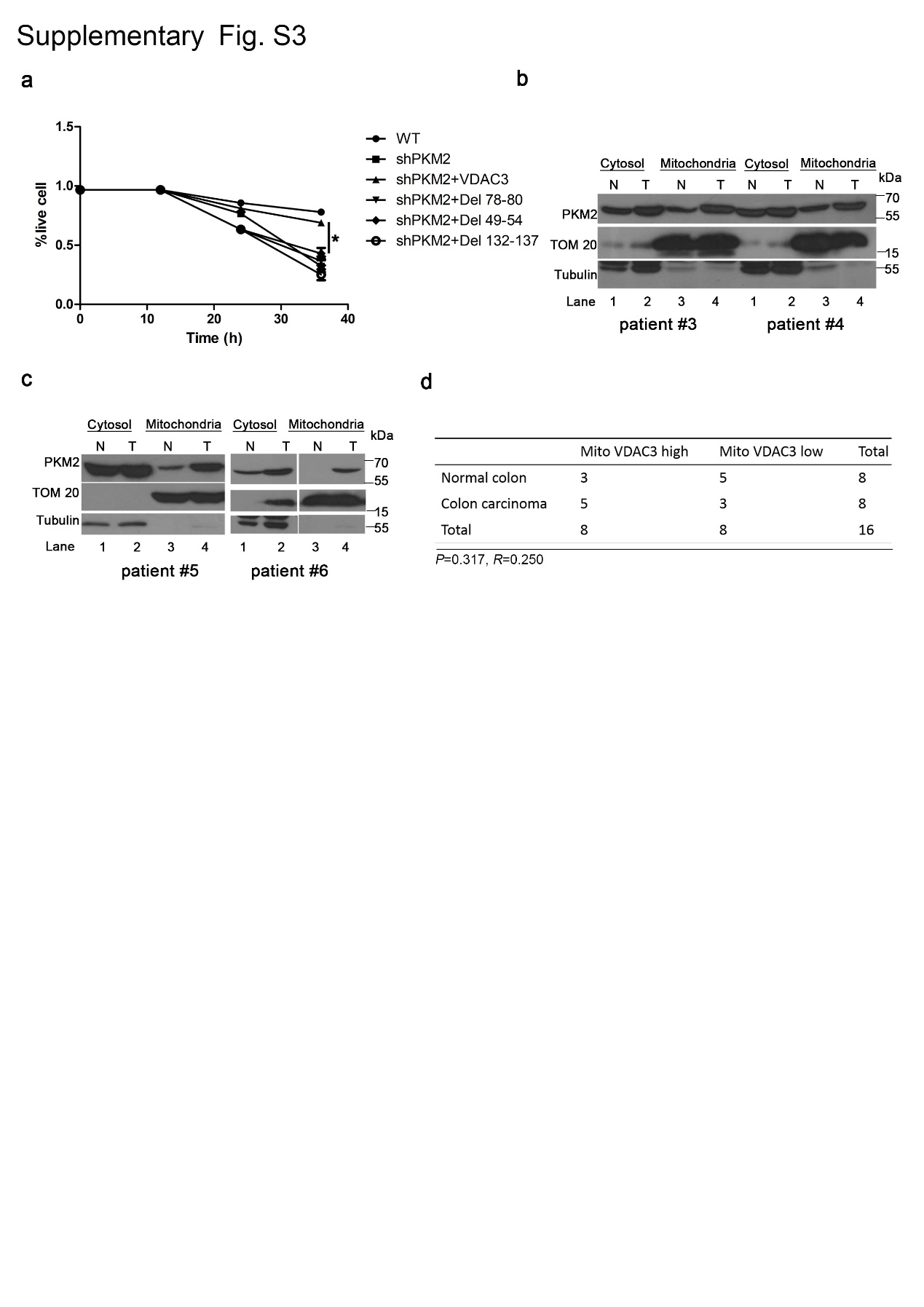


Supplementary Fig. S3. PKM2 and VDAC3 promote cell survival and tumor development under nutrition stress.

**a** PKM2 binding null mutants failed to rescue PKM2 knockdown cell survival under glucose starvation. **b** and **c** Four colon cancer samples that showed enhanced mitochondrial translocation of PKM2. Fresh colon cancer and matched surrounding normal tissue from the same given patient were homogenized, and cytosolic and mitochondria fractions were separated. PKM2 was determined by western analysis. N (normal tissue), T (colon cancer). **d** Statistical table of upregulated VDAC3 in colon cancer samples as compared with adjacent normal tissues. Statistical significance was determined with the χ2-test. R is the correlation coefficient.


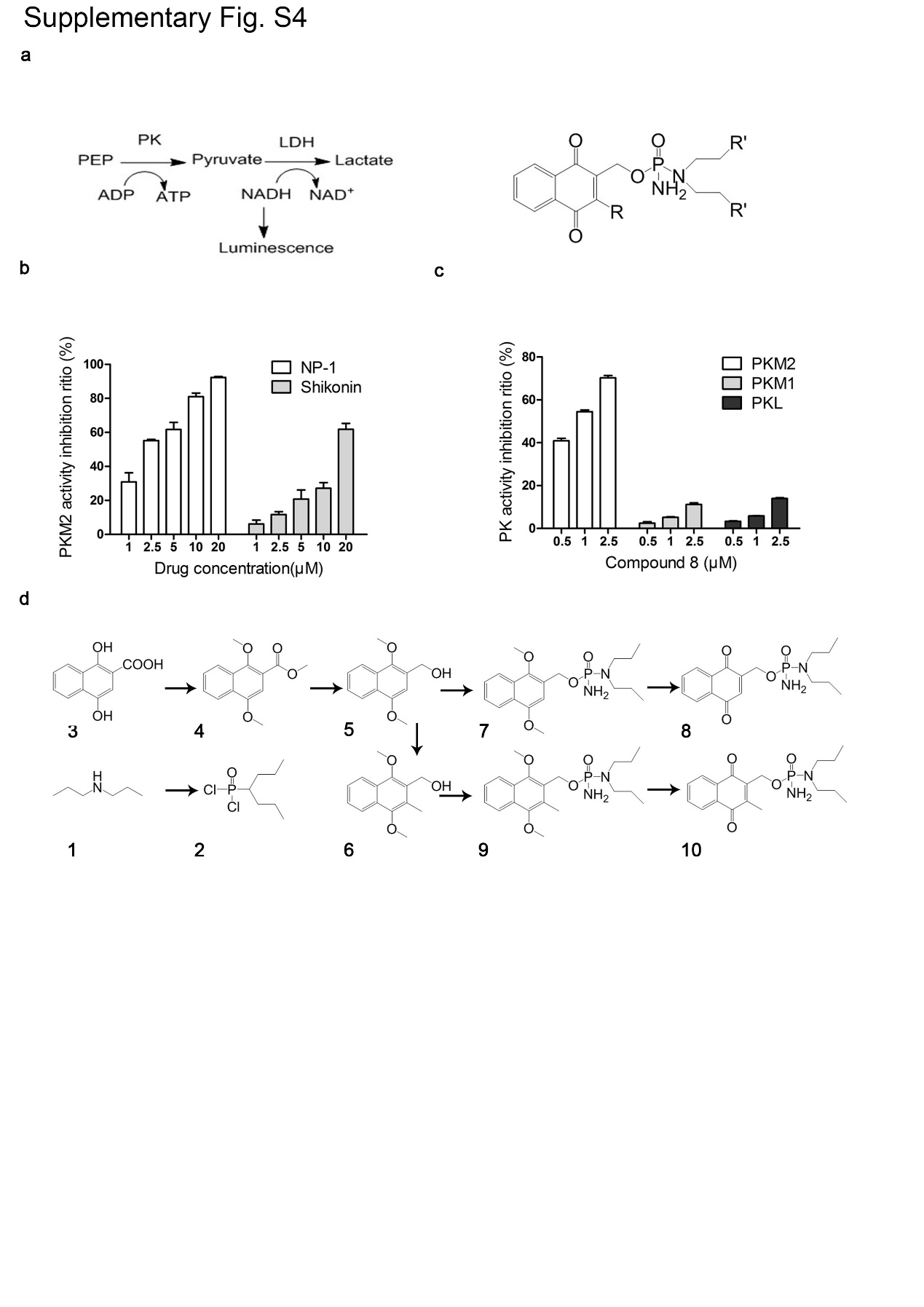


Supplementary Fig. S4. Identification and characterization of small-molecule PKM2-specific inhibitors.

**a** In vitro fluorescent PK-LDH coupled assay used for the primary screen and general structure of compound 8 derivatives. **b** NP-1 and shikonin inhibited PKM2 activity in a dose dependent manner. NP-1 showed greater inhibitory activity than the positive control shikonin. **c** Compound 8 markedly inhibited PKM2, but inhibited PKM1 and PKL to much smaller extent. **d** Synthesis of naphthoquinonephosphorodiamidate derivatives. Reagents and conditions: a) POCl3, NEt3, CH2Cl2, 0°C-rt; b) CH3I, K2CO3, acetone, reflux; c) LiAiH4, THF, reflux; d) CH3I, nBuLi, THF, -78 °C to rt; e) LiN(TMS)2, 2, NH3, THF, -78 °C to -20 °C; f) Ce(NH4)2(NO3)6, CH3CN, H2O, rt.

**Supplementary Methods**

Synthesis of compounds 7 and 9.

Lithium bis(trimethylsilyl) amide (LiHMDS) (3.80 mL, 3.80 mmol, 1.0 M in THF) was added dropwise with a syringe to a solution of compound 5 or 6 (3.44 mmol) in THF (15 mL) at -78 °C under argon. Compound 5 or 6 was synthesized according to a previous report. The resulting solution was stirred for 5 min and added dropwise with a syringe to a solution of phosphorylating agent 2 (900 mg, 4.13 mmol) in THF (20 mL) at -78 °C. The reaction mixture was stirred at -78 °C for 1.5 h and was then warmed to -20 °C. Gaseous ammonia was passed through the reaction mixture for 20 min. The mixture was stirred for 30 min, and 2% aqueous HCl (40 mL) was added and the mixture was extracted with ethyl acetate.The combined organic fractions were washed with brine, dried (Na2SO4), and concentrated under reduced pressure. Purification of the crude residue by column chromatography (petroleum ether/ethyl acetate) afforded compound 7 or 9.

Data for 2-(1,4-Dimethoxynaphthyl)methyl N,N-dipropylphosphorodiamidate (7): white solid; yield of 44%; mp 85−86 °C; 1H NMR (400 MHz, CDCl3) δ 8.25 (dd, 1H, ArH), 8.07 (dd, 1H, ArH), 7.54 (m, 2H, ArH), 6.90 (s, 1H, CH=C-O), 5.20 (m, 2H, CH2O), 4.01 (s, 3H, OCH3), 3.94 (s, 3H, OCH3), 3.06 (m, 4H, 2CH2CH2CH3), 2.66 (bs, 2H, NH2), 1.58 (m, 4H, 2CH2CH2CH3), 0.89 (m, 6H, 2CH2CH2CH3); 12C NMR (100 MHz, CDCl3) δ 152.07, 147.56, 128.37, 126.72, 126.62, 125.81, 124.98, 124.90, 122.42, 122.03, 104.13, 62.97, 61.94, 61.90, 55.70, 47.82, 47.78, 22.05, 11.33; 31P NMR (CDCl3) δ 17.40; HR-MS (ESI+) m/z 381.19423 [M + H]+, found 381.19329 [M + H]+.Data for 2-(3-Methyl-1,4-dimethoxynaphthyl)methyl N,N-dipropylphosphorodiamidate (9): white solid; yield of 60%; mp 132−133 °C; 1H NMR (400 MHz, CDCl3) δ 8.10 (d, 2H, ArH), 7.53 (m, 2H, ArH), 5.35 (m, 1H, CH2O), 5.19 (m, 1H, CH2O), 3.99 (s, 3H, OCH3), 3.90 (s, 3H, OCH3), 3.03 (m, 4H, 2CH2CH2CH3), 2.55 (s, 3H, C=CCH3), 1.58 (m, 4H, 2CH2CH2CH3), 0.89 (m, 6H, 2CH2CH2CH3); 12C NMR (100 MHz, CDCl3) δ 151.81, 150.32, 129.24, 127.07, 126.75, 125.62, 122.86, 122.24, 63.61, 61.33, 59.30, 59.25, 47.74, 47.70, 22.05, 12.19, 11.30; 31P NMR (CDCl3) δ 16.75; HR-MS (ESI+) m/z 395.20997 [M + H]+, found 395.20927 [M + H]+.

Synthesis of compounds 8 and 10.

Ceric ammonium nitrate (0.69, 1.25 mmol) in H2O (5 mL) was added in aliquots over 15 min to a solution of compound 7 or 9 (0.50 mmol) in CH3CN (20 mL). The reaction was stirred at room temperature for 1 h. The reaction mixture was concentrated in vacuo and extracted with CHCl3. The combined organic fractions were washed with brine, dried (Na2SO4), and concentrated under reduced pressure. Purification of the crude residue by column chromatography (petroleum ether/ethyl acetate) afforded compound 8 or 10.

Data for 2-(1,4-Naphthoquinonyl)methyl N,N-dipropylphosphorodiamidate (8): white solid; yield of 52%; mp 131−132 °C; 1H NMR (400 MHz, CDCl3) δ 8.11 (m, 2H, ArH), 7.77 (m, 2H, ArH), 7.06 (s, 1H, CH=C-O), 4.99 (m, 2H, CH2O), 3.07 (m, 4H, 2CH2CH2CH3), 2.74 (bs, 2H, NH2), 1.58 (m, 4H, 2CH2CH2CH3), 0.91 (m, 6H, 2CH2CH2CH3); 12C NMR (100 MHz, CDCl3) δ 184.68, 184.24, 146.64, 146.55, 134.11, 133.86, 133.49, 131.96, 126.38, 60.78, 60.75, 47.81, 47.77, 22.07, 11.30; 31P NMR (CDCl3) δ 17.49; HR-MS (ESI+) m/z 351.14737 [M + H]+, found 351.14666 [M + H]+.

Data for 2-(3-Methyl-1,4-naphthoquinonyl)methyl N,N- dipropylphosphorodiamidate (10): white solid; yield of 65%; mp 87−88 °C; 1H NMR (400 MHz, CDCl3) δ 8.13 (m, 2H, ArH), 7.76 (m, 2H, ArH), 5.03 (m, 2H, CH2O), 3.02 (m, 4H, 2CH2CH2CH3), 2.77 (bs, 2H, NH2), 2.35 (s, 3H, C=CCH3), 1.56 (m, 4H, 2CH2CH2CH3), 0.89 (m, 6H, 2CH2CH2CH3); 12C NMR (100 MHz, CDCl3) δ 185.26, 183.84, 147.65, 140.05, 133.82, 133.74, 132.13, 131.86, 126.50, 126.45, 57.87, 57.82, 47.77, 47.73, 21.98, 12.82, 11.28; 31P NMR (CDCl3) δ 17.52; HR-MS (ESI+) m/z 365.16302 [M + H]+, found 365.16194 [M + H]+.
